# We Want More Than Life-Sustaining Treatment during End-of-Life Care: Focus-Group Interviews

**DOI:** 10.3390/ijerph18094415

**Published:** 2021-04-21

**Authors:** Mirinae Kim, Minju Kim

**Affiliations:** College of Nursing, Dong-A University, Busan 49201, Korea; kimmili9814@naver.com

**Keywords:** focus groups, end-of-life, life support, decision making, advance care planning

## Abstract

We qualitatively investigated end-of-life care needs. Data were collected via focus-group interviews with three groups: young adults, middle-aged adults, and older adults. The key question was, “What kind of care would you like to receive at the end of life?” Interview data were transcribed and analyzed using content analysis. End-of-life care needs were classified into six categories: life-sustaining treatment needs, physical care needs, emotional care needs, environmental needs, needs for respect, and needs for preparation for death. Because the Korean culture is family-oriented and talking about death is taboo, Korean patients at the end of their life do not make decisions about life-sustaining treatment or actively prepare for death. Therefore, to provide proper end-of-life care, conversations and shared decision-making among patients and their families are crucial. Further, we must respect patients’ dignity and help them achieve a good death by understanding patients’ basic care preferences. Future research should continue examining end-of-life care needs that reflect the social and cultural context of Korea to inform instrument development.

## 1. Introduction

With growing societal interest concerning the meaningless use of life-sustaining devices and death with dignity, needs for advanced directives (ADs) or advanced care planning (ACP) are increasing worldwide, including in Korea. Whereas the United States enacted the Patient Self Determination Act in 1990 and the United Kingdom enacted the Mental Capacity Act in 2005, Korea enacted the Hospice, Palliative care, and Life-sustaining Treatment Decision-making Act (HPL Decision-making Act) only in 2018, thereby establishing a system to protect individuals’ right to make end-of-life decisions.

In Korean culture, talking about death is taboo [1], and family-centered decision-making is preferred to individual-centered decision-making about disease treatment and withdrawing life-sustaining treatment [2]. Such cultural features have hindered individuals in making their own decisions about their desired end-of-life care (EOLC). In 2003, prior to the enactment of the HPL Decision-making Act, only 7.3% of individuals made the decision to place a “do not resuscitate” (DNR) order on their own, with most cases of DNR orders decided by patients’ families [3]. However, following the enforcement of the act, approximately 0.4% (~220,000) of the Korean population wrote their ADs between February 2018 and May 2019, and approximately 50,000 people made their own decisions to withdraw life-sustaining treatment, which accounts for 31.8% of the patient population for whom life-sustaining treatment was withdrawn. Although the percentage of patients making their own decisions for withdrawal of life-sustaining treatment is rising rapidly [4], the rate is still markedly low given the fact that about 37% of the American population in 2016 wrote an AD [5]. To protect patients’ right to self-determination while considering the Korean culture in which family-centered decision-making prevails, it is essential to develop ACP by involving patients and their families.

ACP refers to a decision-making process for EOLC for individuals of all ages and health conditions. It is the process by which individuals explore palliative treatment options, exchange opinions about medical treatment, and clarify their preferences and wishes about life-sustaining treatment. During this process, individuals’ care preferences are also discussed [6]. By developing ACP, unnecessary life-sustaining treatment can be reduced, while the patients’ and their families’ satisfaction with EOLC is boosted [7,8,9].

Currently, the HPL Decision-making Act of Korea is focused on life-sustaining treatment provided by healthcare providers, such as cardiopulmonary resuscitation (CPR), mechanical ventilation, hemodialysis, and extracorporeal life support. According to the 2019 statistics on preparation for death among older adults, approximately 43% of the preparations were focused on postmortem care, such as funeral, mutual aid society, burial ground, and shroud [10]. In the United States and Europe, a variety of tools, such as Five wishes or Go Wish cards, have been developed and implemented to examine patients’ diverse care preferences in the physical, psychological, social, and spiritual domains [11]. However, despite the extensive research data examining the preferences for life-sustaining treatment in Korea, studies investigating various EOLC needs in the terminal phase are still lacking. To improve the care provision for patients nearing the end of their lives and their families, a Korean version of ACP that encompasses patients’ various care needs is essential [12]. To this end, the types of care desired by patients needing EOLC should be identified.

Therefore, we conducted a comprehensive and in-depth analysis of EOLC among community residents via focus-group interviews (FGIs). The benefits of an FGI include the ability to obtain abundant data focused on EOLC needs, which cannot be obtained simply via observation and the ability to obtain data from various perspectives through interactions among group members.

## 2. Materials and Methods

### 2.1. Study Design

We employed a qualitative descriptive design, which offers a comprehensive summary of events in everyday language [13,14].

### 2.2. Participants

Participants were community residents who expressed interest after seeing advertisements for the study at community facilities (e.g., public health offices, town halls, colleges) in Busan, Korea. Moreover, snowball sampling was used to recruit participants until the process generated no new references. Inclusion criteria were (1) age > 20 years and (2) understanding the study purpose and agreeing to participate. As a guiding principle in qualitative research, the sampling was conducted until data saturation was achieved and recommended three to four focus group interviews for content analysis [15]. In this study, nine focus groups were created.

### 2.3. Data Collection

This study was approved by a university’s research board. Thirty-seven participants provided written, informed consent and completed a short demographic questionnaire (Table 1).

Each focus group comprised 3–6 participants, grouped by age in consideration of generational differences concerning death and EOLC. Thus, 3 FGIs included participants in their 20s; 3 FGIs included participants in their 30s, 40s, and 50s; and 3 FGIs included participants in their 60s or older. The same open questions were used in all groups. The main question was, “What kind of care would you like to receive at the end of life?” Several probing and follow-up questions were used to explore any new major themes that arose. FGIs lasted 1–2 h and were audiotaped with the permission of participants. Any nonverbal data were also noted by the researcher.

### 2.4. Data Analysis

Focus-group data were transcribed and imported in NVivo 12 (QRS International Pty Ltd., Chadstone, VIC, Australia) for management and coding. Qualitative content analysis was used to analyze data [14]. Two Korean researchers independently performed the coding process based on Bengtsson’s content analysis approach. Both researchers had multiple academic trainings about qualitative analyses, and one of them had several experiences of analyzing qualitative data. Each researcher read data and made initial codes independently [16]. Each researcher read all focus-group data to get a sense of the whole and identified units’ meaning while concurrently making initial codes. The initial codes were then compared and discussed to organize concepts. Categories and theme clusters were identified and defined through a discussion between researchers. Field notes for nonverbal data were compared with the verbal responses for each category [16].

## 3. Results

### 3.1. Sample Description

The study involved 9 focus group interviews with 37 participants. Table 1 shows the participants’ characteristics.

### 3.2. Thematic Analysis

Seventy-three concepts under twenty-one thematic clusters and six categories emerged (Table 2). The six categories were as follows: needs for life-sustaining treatment, needs for physical care, needs for emotional care, environmental needs, needs for respect, and needs for preparation for death at end of life.

### 3.3. Category 1: Needs for Life-Sustaining Treatment

- Theme cluster 1: Main decision-maker for life-sustaining treatment

Participants stated that they were the main decision-makers for withdrawing life-sustaining treatment and they can inform their families of their decisions to withdraw life-sustaining treatment and document it. However, they mentioned that, in their experience, life-sustaining treatment can be performed regardless of their wills, mostly because the plans for life-sustaining treatment are made by patients’ families. Further, some participants mentioned that because they lack the medical knowledge and judgment to make decisions regarding life-sustaining treatment, they would have to rely on the judgment of their healthcare providers. Participants further stated that patients’ families should decide to withdraw life-sustaining treatment to allow the patient to die peacefully; however, they also mentioned that families may regret their decision later.

*“Children can’t just give up on their parents…I should tell my kids that if mom and dad suddenly collapses, we don’t want life-sustaining treatment”*

(older adult 6).

- Theme cluster 2: Factors involved in choosing life-sustaining treatment

Many participants mentioned refusing life-sustaining treatment depending on the situation, and they said that they would refuse life-sustaining treatment in situations such as “when CPR is needed,” “when they are unconscious,” “when the treatment is meaningless and painful,” and “when the treatment is invasive.” Further, participants stated that they would refuse specific treatments such as “endotracheal intubation,” “tracheostomy,” and “mechanical ventilation”; while some stated that they want active euthanasia if they are institutionally permitted. Furthermore, some stated that they would refuse life-sustaining treatment for the sake of their family, primarily owing to the psychological and financial burden it will impose on them.

*“Drilling a hole in your throat—that’s not really what I want. There is no use to just sustain your life like that”*

(middle-aged adult 4).

*“People especially don’t want to become a burden when they become old…they want to leave at least a little money and not become dead weight. It’s the same for everyone”*

(middle-aged adult 9).

- Theme cluster 3: Desired healthcare at end of life

While most participants refused life-sustaining treatment for EOLC, some wished to receive medical treatments such as “pain control,” “sleep improvement,” “nutritional supplements,” and addressing “bowel problems” for their physical and mental comfort. Some stated that they will choose hospice care.

*“…They’re not gonna live for a year or two; they just have to live a day or two, huh? It just doesn’t work ’cause it’s so painful”*

(older adult 6).

### 3.4. Category 2: Needs for Physical Care

- Theme cluster 1: Maintain a good shape

Participants wished to maintain a good external appearance. Specifically, they wished to be physically comfortable without any bedsores or bodily injuries and without being surrounded by medical devices. Receiving clean clothes and bedding was important to them.

*“I just wish to have all the basic necessities taken care of. Just clean me often and change my diapers often”*

(middle-aged adult 5).

*“They don’t change diapers often…I hate bedsores! I’d want to die if I have that”*

(middle-aged adult 6).

- Theme cluster 2: Wish to be protected

Participants wished to be protected from bodily exposures and remain safe from physical dangers such as falls. Some even positively perceived the use of physical restraints to ensure safety.

*“You fall from your bed and get fractures in the blink of an eye…So you need to be tied up [with physical restraints] if you don’t want to be hurt”*

(older adult 12).

- Theme cluster 3: Dynamic activities

Some participants mentioned that they want to engage in dynamic activities, such as taking a stroll, going outdoors, doing exercise, and getting a massage to move their bodies as they would normally do in their daily lives instead of remaining bedridden.

*“I have no intent to remain in a ward and lying down on a bed. I just want to go outside, even just a little bit, when I am still capable of walking on my feet and get some fresh air”*

(older adult 12).

- Theme cluster 4: Personally preferred physical care

Participants mentioned “eating food and having drinks,” “enjoying indulgences,” and “enjoying hobbies” as their personally preferred physical care.

*“Even if eating this would be bad for my health, I want it when I like it at the moment. I’m going to die anyway”*

(middle-aged adult 6).

### 3.5. Category 3: Needs for Emotional Care

- Theme cluster 1: Supportive care to protect against the fear of death

Some participants stated that they wanted to receive emotional support that would relieve their anxieties about death.

*“I wish they would let me know that I’m not alone. I wish they would help me feel relaxed. So that I won’t be nervous. So that I could depend on them”*

(young adult 4).

*“Thinking that I will die. I think I’ll be so lonely. I think I’m going to be really lonely; so, I wish my close friends would visit me often to say, ‘hi’”*

(middle-aged adult 7).

- Theme cluster 2: Religious care

Some participants wanted to rely on religion, be psychologically comforted, and be prepared to face death through religious activities such as “praying,” “singing,” and “being visited by religious personnel.”

*“No matter if you have a religion or not, you get to look back at your life when you die. People suddenly confess their sins…For example, If I believe (in God) but my mom doesn’t, then when the pastors come to my mom nearing her death then she suddenly develops faith. Because they tell you that you will go to heaven when you die”*

(young adult 9).

*“I transcribed the entire Bible, and I wish that someone would bring that notebook and read it for me—even if I’m not conscious”*

(older adult 1).

### 3.6. Category 4: Environmental Needs

- Theme cluster 1: Independent space

Participants stated that they want to spend the final days of their lives in an independent space to ensure their privacy and not be stirred by surrounding environments or other patients.

*“I think it would be difficult on me to see the whole ward be depressed and sad because of my death. Everybody here is sick, and when they become sad, they can be even more depressed…”*

(young adult 9).

*“I don’t want to have to be forced to get along with other people…I wish that people have their private spaces and just gather when we have to”*

(middle-aged adult 5).

- Theme cluster 2: Familiar environment

Most participants wanted to spend the final days at home instead of dying at the hospital.

*“I wish I would spend the later days of my life at a place where I lived for a long time and where I have all the memories. I want to go back to my hometown and remind myself of the memories and not stay in the hospital…”*

(young adult 10).

- Theme cluster 3: Personally preferred environment

Some participants stated a place that can be often visited by their families or friends as an essential environmental factor for EOLC. The specifically preferred environmental needs varied depending on individuals’ tendencies.

*“When you’re lying down, the people you miss the most are your grandchildren and immediate family…”*

(older adult 9).

*“I wish it would be a place where people can often come, and friends can come and go”*

(young adult 6).

- Theme cluster 4: Want to choose one’s care provider

Participants wished to spend much of their time with their family during EOLC. Some mentioned that they would refuse the care of people other than their families owing to the embarrassment of exposing their bodies and the difficulties of comfortably expressing their desired care needs to the care providers at hospitals or long-term care facilities. In contrast, some wished to be cared for by “healthcare providers,” “professional care providers,” and “intimate others” instead of their family. Reasons included the physical and financial burden imposed on family members and wanting to rely on healthcare providers’ expertise.

*“Nurses or care provider[s] can wash me and change my diapers; but I think it would be difficult for them to do that for me without making me feel embarrassed. So, I wish my family would do that for me if I am conscious at all”*

(young adult 2).

*“The problem is whether they treat patients who are admitted [to the hospital] nicely. You never know how they [care providers] will treat patients”*

(middle-aged adult 7).

### 3.7. Category 5: Needs for Respect

- Theme cluster 1: Respectful treatment

Participants wanted to be treated with dignity at the end of their lives and in a polite and friendly way. Some stated that they did not want others to over-discuss their private lives or disease.

*“I wish the healthcare providers wouldn’t be so cold…”*

(older adult 13).

*“I don’t want people around me to be interested in me so much. It’s enough that I worry about death and if I make people around me worry. They have their own lives; so, I want them to be polite but not care about me too much”*

(young adult 3).

- Theme cluster 2: Physical respect

Participants stated that they wanted respect from their care providers regarding bodily exposure or injuries. In addition, some participants mentioned the problem of abuse in long-term care facilities.

*“I don’t want people to see me like that…If death is near, you only have your bones left. You look like a ghost and not a grandfather”*

(older adult 9).

### 3.8. Category 6: Needs for Preparation for Death

- Theme cluster 1: Accept death

Some participants mentioned that they want to spend the final days of their lives meaningfully by accepting their death and reminiscing about their life and memories. Some stated that, by accepting death like this, they can prepare for the separation from their family.

*“Practice to end bonds with life and children, I want to practice [those] kind[s] of things”*

(middle-aged adult 5).

*“I want to go in my bed, my eyes closed, and be farewelled by my family. I want to go when it is time…I want to see death as a joyful thing and don’t want to fear it”*

(young adult 4).

- Theme cluster 2: Meeting and talking

Participants stated that they want to meet people they wish to see before they die to talk with them, say goodbye, deliver messages of hope and love, and ask for forgiveness.

*“I’m just curious how my family, friends, and people around me would judge me after I die…The Korean culture is not like where you talk about everything with your family”*

(middle-aged adult 6).

*“I think it would be too much of a burden if I ask before I die. I want to say things like, ‘I was happy to be with you.’ ‘Don’t be sad’”*

(middle-aged adult 5).

- Theme cluster 3: Remember me

Participants wanted people to have good memories about them after they are gone. Some said they will use channels such as videos, wills, letters, memoirs, and autobiographies. Further, they wanted the news of their death to be delivered to those who cannot visit them.

*“If I have a care provider, I wish they would tell people who visit me that I am at peace even if I’m not conscious. I wish they would tell people not to worry or be sad. And even if some family members couldn’t stay beside me at my deathbed, I wish people would tell them that I went in peace because they could feel sad—at least at the funeral”*

(middle-aged adult 7).

- Theme cluster 4: Have affairs in order

Most participants referred to their preparation for death as “having your affairs in order.” They wanted to clean up their stuff in the room or house as well as their phone numbers and emails.

*“I wish they would clean up my house or room”*

(middle-aged adult 6).

*“I think I would deal with my cell phone first. Tell my family the password. Let them know who to contact”*

(middle-aged adult 7).

- Theme cluster 5: Arrange for postmortem affairs

Participants wanted to plan for their funerals and notify others of their wishes. Some older participants had already prepared their own portraits and cemeteries.

*“I should be cremated. I want that and I don’t need any memorial ceremonies or what not”*

(older adult 3).

## 4. Discussion

We conducted FGIs with the residents of communities in Korea to identify specific EOLC needs. Six categories were identified: “needs for life-sustaining treatment,” “needs for physical care,” “needs for emotional care,” “environmental needs,” “needs for respect,” and “needs for preparation for death.”

The “needs for life-sustaining treatment” category included statements about the main decision-makers for life-sustaining treatment and desired medical care at the end of life, which shed light on the perception and problems of life-sustaining treatment among Koreans. The result that participants were aware of their right to self-determination in life-sustaining treatment was consistent with the results of Western studies [17]. However, in the present study, main decision-maker for life-sustaining treatment was divided into self and family, indicating participants were adequately aware of their right to self-determination regarding end-of-life life-sustaining treatment but that they believed that life-sustaining treatment that is against their wills can be provided. In the family-centered decision-making culture in Korea, decisions about life-sustaining treatments have conventionally been made by the family [2,18]. Therefore, there is a need for opportunities and communication channels to properly discuss the EOLC needs of patients and their families, and healthcare providers should actively involve patients and their families in these processes [19]. Shared decision-making involving patients, families, and healthcare providers may alleviate patients’ and families’ psychological burden of having to make a choice [20]. Further, participants presented vague and comprehensive expressions regarding the timing of withdrawing life-sustaining treatment, such as “when I am unconscious,” “when I have difficulty breathing,” and “when I feel that life-sustaining treatment makes my death miserable.” In fact, the timing of withdrawing life-sustaining treatment as delineated in Korea’s HPL Decision-making Act is for patients nearing the end of life; however, the terms “end of life” and “terminally ill” are only theoretical concepts that represent the differences in the residual lifespan and, thus, are difficult to operationalize in reality.

The “needs for physical care” category illustrates that patients wished to autonomously express their physical care needs according to their physical states and personal preferences. Most stated that they want to maintain “good shape” and look presentable. Having an undesirable, negative bodily appearance can seriously undermine one’s self-esteem [21]. In addition, participants wanted to be provided with basic care, such as bathing and having clean clothes and bedding. Thus, withdrawal of life-sustaining treatment does not mean that basic care needs should be forfeited; each individual’s care needs should be identified and satisfied through proper communication skills [22].

Participants also stated the need for physical protection under the category “needs for physical care,” which was defined as the protection of personal privacy, such as preventing bodily exposure and protection from physical harms or risks. Many positively perceived the use of body restraints for this purpose. This is contradictory to recent study findings [23,24] that the use of body restraints should be diminished and the reasons for using body restraints should be analyzed first, as body restrains provoke ethical problems and do not necessary ensure patient safety. Because our participants’ reason for consenting to the use of body restraints was to protect themselves from physical risks and since patients were experiencing physical and cognitive functional decline, additional studies are needed to investigate the needs for body restraints among patients requiring EOLC.

The “needs for emotional care” category showed that participants wished to be given supportive care that relieves their fear of death and religious care that prepares them for death. The pain of patients nearing the end of life encompasses physical, psychological, and spiritual aspects, which are all closely related to one another [25]. Patients may experience various negative emotions, such as fear, anger, sorrow, tension, and loneliness [26], and it is important to carefully listen to their stories and express empathy and support. Participants wished for nonverbal communication, which is in line with the finding that nonverbal communication such as making eye contact and holding hands is perceived as positive emotional support [27]. However, although nonverbal communication between patients and healthcare providers helps strengthen their trust relationship, studies analyzing nonverbal communication between actual patients nearing the end of life and their healthcare providers are lacking, calling for studies investigating appropriate nonverbal communication methods and education.

The “environmental needs” category comprised the theme clusters independent space, familiar environment, personally preferred environment, and ability to choose care provider. Participants wanted to have an independent space at the end of life because they wanted their privacy to be protected and also a place to rest while avoiding the tense environment around them. In contrast, under the theme cluster “personally preferred environment,” participants wanted to be in a place that can be often visited by their families or friends, showing contradictory environmental needs. In other words, while patients nearing end of life want to be cared for and rest in an independent living space, they also want to maintain relationships. In a study that investigated the factors needed to achieve death with dignity among adults in Korea, being with family or significant other was identified as a vital factor [28]. Further, people receiving hospice care in Korea prefer a four-patient ward to a single-patient ward to alleviate their loneliness [29]. Thus, when providing EOLC, patients should be offered an independent space but also be allowed to be visited by families and friends.

Regarding the ability to choose one’s care provider, some participants wanted to enjoy the comfort provided by their family, while other participants refused care provided by their family but instead wished to receive care from professionals. As reported in a previous study [30], participants were concerned with the challenges of communicating with healthcare providers. Thus, a trust-based relationship and appropriate communication between care providers and patients are important to understand patients’ personal care preferences.

The “needs for respect” category comprised personal respect and physical respect, showing that participants want to be respected and revered as they near death. Participants also wanted to be guaranteed personal privacy at their end of life, such as avoiding body exposure and having personal space; however, some participants mentioned the problem of abuse in long-term care facilities. This pertains to basic human dignity, and the World Health Organization and many countries have developed various measures to address abuse in long-term care facilities [31]. A recent Korean study found that the level of emotional labor among personnel providing EOLC for patients was associated with increased depersonalization [32]. Thus, systematic education is needed to assist EOLC-providing staff in showing respect for patients, and measures to reduce staff burnout and emotional labor should also be investigated. Such measures would help EOLC-providing personnel identify and address patients’ needs and improve their care provision.

The “needs for preparation of death” category comprised theme clusters about preparing for death, which reduces individuals’ anxieties about death while promoting spiritual well-being and meaning of life. However, death is a taboo topic in Korean culture, which may elevate people’s fear of death [33]. Patients receiving EOLC should be encouraged to express their thoughts about death and mentally prepare for death. It is necessary to develop an instrument, such as “Five wishes,” which is one of the ADs (legally permitted in 44 states) in the USA [34], to encourage Korean individuals to make choices and practically prepare for their death, including matters related to life-sustaining treatment.

In this study, self-determination in making decisions about EOLC was important in all age groups. Middle aged and elderly groups talked about their preferences for specific treatment methods based on their experience with death and dying, while young adults focused on emotion and psychological care. Further study is needed to examine the differences in EOLC needs by age group. In addition, it is necessary to develop communication tools that make it easy to share one’s EOLC needs with their loved ones. There is a limitation to generalizing the results to other populations because this study was conducted a city in Korea. Therefore, it is necessary to considering diversifying the research subjects’ regions and sociodemographic groups for future research.

## 5. Conclusions

We conducted FGIs and content analyzed the data to identify EOLC needs. Notably, we presented EOLC strategies in accordance with the social and cultural contexts of Korea, where death is a taboo topic of conversation. Thus, in Korea, sufficient discussion and shared decision-making are essential between patients receiving EOLC and their families. Although there is a limitation to generalizing the research results, this study shed light on the fact that these patients require basic care such as cleanliness, safety, respect, and rest, in addition to care related to life-sustaining treatment. Hence, in addition to respecting patients’ human dignity, stakeholders must identify their care preferences to help them maintain a good quality of life at the end of life. Finally, we hope that researchers continue to examine EOLC needs that reflect Korea’s social and cultural contexts and that studies develop instruments to effectively measure EOLC needs based on such findings.

## Figures and Tables

**Table 1:** Participants’ characteristics by age group.

Characteristic		Young (*n* = 10)	Middle (*n* = 12)	Older (*n* = 15)
Age (years)	Mean (SD)	24.6 (1.6)	46.0 (10.3)	65.5 (3.5)
Sex	Male	3	4	4
	Female	7	8	11
Employment status	Employed	0	10	4
	Unemployed	10	2	11
Marital status	Single	10	6	0
	Married	0	6	15
Religion	None	4	5	5
	Religious	6	7	10

**Table 2:** Main categories and theme clusters.

Categories	Theme Clusters
Needs for life-sustaining treatment	Main decision-maker for life-sustaining treatment
	Factors involved in choosing life-sustaining treatment
	Desired healthcare at end of life
Needs for physical care	Maintain a good shape
	Wish to be protected
	Dynamic activities
	Personally preferred physical care
Needs for emotional care	Supportive care to protect against the fear of death
	Religious care
Environmental needs	Independent space
	Familiar environment
	Personally preferred environment
	Want to choose one’s care provider
Needs for respect	Respectful treatment
	Physical respect
Needs for preparation for death	Accept death
	Meeting and talking
	Remember me
	Have affairs in order
	Arrange for postmortem affairs

## Data Availability

Data available on request due to restrictions, e.g., privacy or ethical.
